# Training with an auditory perceptual learning game transfers to speech in competition

**DOI:** 10.1007/s41465-021-00224-5

**Published:** 2021-09-21

**Authors:** E. Sebastian Lelo de Larrea-Mancera, Mark A. Philipp, Trevor Stavropoulos, Audrey Anna Carrillo, Sierra Cheung, Tess K. Koerner, Michelle R. Molis, Frederick J. Gallun, Aaron R. Seitz

**Affiliations:** 1 Psychology Department, University of California, Riverside, Riverside, CA USA; 2 Brain Game Center, University of California, Riverside, Riverside, CA USA; 3 Oregon Health and Science University, Portland, OR USA; 4 VA RR&D National Center for Rehabilitative Auditory Research, Portland, OR USA

**Keywords:** Perceptual learning, Auditory training, Cognitive training, Video-games, Supra-threshold auditory function

## Abstract

Understanding speech in the presence of acoustical competition is a major complaint of those with hearing difficulties. Here, a novel perceptual learning game was tested for its effectiveness in reducing difficulties with hearing speech in competition. The game was designed to train a mixture of auditory processing skills thought to underlie speech in competition, such as spectral-temporal processing, sound localization, and auditory working memory. Training on these skills occurred both in quiet and in competition with noise. Thirty college-aged participants without any known hearing difficulties were assigned either to this mixed-training condition or an active control consisting of frequency discrimination training within the same gamified setting. To assess training effectiveness, tests of speech in competition (primary outcome), as well as basic supra-threshold auditory processing and cognitive processing abilities (secondary outcomes) were administered before and after training. Results suggest modest improvements on speech in competition tests in the mixed-training compared to the frequency-discrimination control condition (Cohen’s *d* = 0.68). While the sample is small, and in normally hearing individuals, these data suggest promise of future study in populations with hearing difficulties.

**Supplementary Information:**

The online version contains supplementary material available at 10.1007/s41465-021-00224-5.

## Introduction

Despite a vast amount of research conducted across multiple fields, clinicians and researchers still disagree about the best ways to confront the full diversity of hearing difficulties individuals face throughout their lives. Historically, auditory rehabilitation has been focused on the ability to *detect* sounds—audibility. To this end, hearing loss due to elevation of auditory detection thresholds can often be addressed through the use of amplification technologies such as hearing aids (Chisolm et al., 2007). However, although hearing aids can restore at least partial audibility for some listeners, even in the presence of competing sounds (Humes et al., 2009), and are increasingly recommended for those with hearing complaints associated with central auditory processing (CAP) dysfunction (Koerner et al., 2020), there is little documented clinical evidence supporting the prescription of hearing aids for those with pure-tone detection thresholds in or near the normative range for young adults. Moreover, amplification technologies may actually present difficulties in noisy environments since both sounds of interest and competing background noises are amplified together with no relative increase in the audibility of the signal.

Similarly, conventional hearing aids may not provide the best solution for those with supra-threshold auditory processing difficulties, which often manifest as a reduced capacity to *discriminate* among competing sounds and hinders ones ability to separate auditory signals of interest from competing background noises: for example, individuals with supra-threshold auditory processing difficulties may struggle to understand one voice out of a group of many talkers even when sounds are audible (above hearing threshold). The more general case of this difficulty of hearing in multiple talker environments is often referred to as the cocktail party effect (Cherry, 1953; McDermott, 2009). Because currently there are no widely-accepted methods to assess and treat supra-threshold auditory processing difficulties, there is a significant need for novel approaches to evaluate and rehabilitate these common hearing complaints (Gallun et al., 2014, 2018; Hoover et al., 2017; Larrea-Mancera et al., 2020; Weihing et al., 2015).

Auditory training (AT) has been proposed as a promising rehabilitation approach for individuals experiencing hearing difficulties associated with supra-threshold auditory processing (Chermak & Musiek, 2002; Moore & Amitay, 2007; Weihing et al., 2015), including those already using hearing aids for sound amplification (for review see Henshaw & Ferguson, 2013; Stropahl et al., 2020). There is an extensive literature on AT employing a variety of training targets applied to a variety of target populations (see Ferguson & Henshaw, 2015). Training targets have ranged from simple frequency discriminations (Goldsworthy & Shannon, 2014) to phonemes (Ferguson et al., 2014; Kimball et al., 2013; Wade & Holt, 2005), modified speech (Merzenich et al., 1996; Tallal et al., 1996), speech in noise (Burk et al., 2006; Humes et al., 2014; Kuchinsky et al., 2014), active conversation listening (Lavie et al., 2013), and music (Schellenberg, 2016; Zendel et al., 2017). Target populations have included children with learning difficulties (Merzenich et al., 1996; Tallal et al., 1996), cochlear implant users (Goldsworthy & Shannon, 2014), young adults with normal hearing (Kimball et al., 2013; Wade & Holt, 2005; Whitton et al., 2014), older adults both with normal hearing (Karawani et al., 2016; Zendel et al., 2017), and those with hearing difficulties (Anderson et al., 2013a, 2013b; Henshaw & Ferguson, 2013; Whitton et al., 2017; Stropahl et al., 2020). However, the key limitation of many of these training studies is the lack of significant and lasting transfer of learning beyond the trained context (Seitz, 2017).

The goal of the current study is to test a novel approach to auditory training that targets multiple dimensions of hearing with the goal of achieving transfer to supra-threshold processing abilities such as the ability to recognize speech in competition. We adopt a novel “gamified” AT approach that integrates training principles from two main fields of knowledge: perceptual learning (PL; see Seitz, 2017) and video-game play (see Bavelier et al., 2009).

In PL, transfer of learning to untrained stimulation or conditions has been shown after repeated training with perceptual stimuli when 1) the task is neither too hard nor too easy (Ahissar & Hochstein, 1997; Ghose et al., 2002; Hung & Seitz, 2014), 2) training includes a diverse stimulus set (Deveau et al., 2014a, b; Xiao et al., 2008; Zhang et al., 2011), 3) exogenous (Donovan et al., 2015) or endogenous attention is directed towards trained cues (Donovan & Carrasco, 2018), and 4) more than one sensory modality guides participant interactions with the training stimuli (Shams & Seitz, 2008; Shams et al., 2011).

Gamification is motivated based on findings that some commercial video games lead to broad improvements across a number of visual and cognitive processing skills (Bavelier et al., 2009; Bediou et al., 2018; Green & Bavelier, 2003). However, careful integration and design of game-elements is essential as game elements can also be distracting and interfere with learning (Katz et al., 2014; Mohammed et al., 2017see also Seitz et al., 2010). Furthermore, games do not always focus performance on the intended processes. For example, Stewart et al. (2020) showed an advantage for action video-game players in visual but not auditory attention, and there was no difference on measures of speech-in-competition ability. Of note, even when auditory cues are useful in so-called “action video-games,” they rarely are essential for solving the tasks or maximizing outcomes, which may explain why visuo-spatial skills are more likely to be trained than are auditory skills.

Previous work at the University of California, Riverside Brain Game Center for Mental Fitness and Well-being (BGC) has successfully integrated the framework of PL with commercial video-game principles in the visual domain (Deveau & Seitz, 2014; Deveau et al., 2014a, b; Deveau et al., 2014a, b). Deveau and colleagues developed a game where the goal was to quickly find oriented line patterns (Gabor patches) that varied on a number of stimulus dimensions to train vision. The authors found that systematic training across visual primitive features such as the spatial frequencies, orientations, and locations of presentation of classic low-level visual stimuli (Gabor patches), with adaptive difficulty on detectability of the stimuli, resulted in broad transfer of learning across basic tests of vision (Deveau et al., 2014a, b), reading (Deveau & Seitz, 2014) and even to on-field performance in baseball athletes (Deveau et al., 2014a, b).

Crucial to this approach was the use of stimuli that align with primitive features found to be systematically represented in the early sensory cortices (Hubel & Wiesel, 1962, 1968) and in particular their sufficiency as a basis set (e.g., spanning a set of dimensions that in combination can represent any stimulus in a particular stimulus space). For example, in the case of vision, a set of filters that span dimensions of spatial frequency, orientation and spatial location are mathematically sufficient to represent any image (ignoring color). This represents a core concept in our approach, that training based upon a basis set of perceptual dimensions that are sufficient to represent the perceptual space of interest would provide a principled approach to obtain transfer of learning to the broad range of stimuli described by that space of features (Seitz, 2018).

This project tests the hypothesis that improvements in supra-threshold auditory processing, including speech in competition, will result from training with the basic perceptual features or processes from which they arise. One challenge in this endeavor is to identify the critical dimensions across tasks and stimuli that are sufficient as a basis function of central auditory processing relevant to speech in competition (Seitz, 2018). Of note, here we are focusing on primitive features that underlie the extraction of speech sounds from competing sources, and are not targeting higher level processes related to the representation of speech itself. One of the most promising sets of candidates for basis functions are spectral and temporal amplitude modulations. There is substantial evidence that these both describe response properties of neurons in the auditory cortex (Kowalski et al., 1996; Shamma, 2001) and can computationally be used to represent any auditory stimulus within a time-spectrum space. Furthermore, spectro-temporal processing ability has also been shown to predict speech intelligibility in individuals who have difficulties both in detecting pure-tones and in understanding speech in quiet and in noise (Bernstein et al., 2013; Mehraei et al., 2014). Based upon this literature, a first set of candidate dimensions for training are spectral-temporal modulation (STM) processing at a variety of frequency ranges, direction, and modulation duration.

Another potential dimension that may form an essential basis set, crucial for auditory scene analysis and for speech in competition, is the information underlying the ability to localize sounds in the environment. Spatial hearing can help segregate information coming from sound targets including speech and reduce the interference caused by distractors at different locations (Gallun et al., 2013). The ability to benefit from spatial hearing cues declines with increases in age and/or in pure-tone detection thresholds (Füllgrabe et al., 2015; Gallun, 2021) and so it represents another candidate for systematic variation.

Additionally, the ability to process sounds in memory (auditory working memory) is an important mediator of auditory learning (Zhang et al., 2016) that is essential to the recognition of speech in competition (Gallun & Jakien, 2019) as well as the listening effort associated with complex acoustical conditions (Peelle, 2018). Previous work has shown that working memory demands are effective at infusing cognitive challenge into perceptual tasks that may in turn promote learning (Bavelier et al., 2009; Green & Bavelier, 2015).

In sum, based on neuroscientific and behavioral grounds, we selected fundamental dimensions of auditory processing that individually and collectively contribute to the ability to listen successfully to speech targets in competition. These were presented in a gamified setting with adaptive difficulty in tasks that focused training on stimulus duration, STM slope, modulation depth, spatial offset, or auditory memory load. Training included resolving these stimuli from competing noise sources, which further allowed task difficulty to be adapted across an ecologically valid dimension (McDermott, 2009). While there have been previous studies that have used video-game elements with AT (Kimball et al., 2013; Tallal et al., 1996; Vlahou et al., 2012; Wade & Holt, 2005), these typically trained on more limited stimulus sets. A notable exception is Whitton et al. (2017), who used a gamified approach that trained older adults with hearing loss on pitch, level, amplitude modulation and speech sounds and found learning transfer to measures of speech in competition (Whitton et al., 2017). Still, research examining how training with a wide range of psychoacoustical and cognitive tasks may lead to improvements in listening to speech in competition is limited and there is a need to examine how training on a theoretically-motivated basis set of basic auditory features may or may not lead to the broad based learning outcomes that have been seen in the case of vision (Deveau et al., 2014a, b).

In this study, the effectiveness of this gamified mixed-training approach was examined in a population of college-aged adults with no reported hearing difficulties. This AT training program, called *Listen* (https://braingamecenter.ucr.edu/games/listen-an-auditory-training-experience/), was developed at the BGC, can be run on mobile devices (e.g., iPad, iPhone, Android) or standard desktop computers (MacOS, Windows) and is currently freely available through the Apple App Store, the Google Play Store, and the Microsoft Store. The AT implemented in *Listen* “gamifies” auditory perceptual tasks into an “endless runner” type of video-game in which the player makes judgements based on spectro-temporal modulations, spatialized sound cues, and previously presented sounds stored in working memory to avoid obstacles and progress within the game environment. Correct and incorrect responses have direct and immediate influence on the adaptive parameters of the game, which we hypothesize will have powerful PL consequences.

To evaluate the effectiveness of this gamified AT approach, we examined the primary outcome of transfer to speech in competition, and then secondary outcomes of transfer to measures of central auditory and cognitive processing before, in the middle (in the case of speech in competition) and after training, with one month follow-up (again only speech in competition). For hearing assessments, we used the Portable Automated Rapid Testing (PART) app (https://braingamecenter.ucr.edu/games/p-a-r-t/), which we have previously demonstrated is capable of reproducing precise acoustic stimuli outside of a controlled lab environment (Gallun et al., 2018) and have validated its performance with a group of college-aged participants with no known hearing difficulties in conditions of moderate environmental noise (Larrea-Mancera et al., 2020). The mixed-training approach was compared to an active control condition comprised of pure-tone frequency discrimination training presented in the same gamified framework but lacking most of the elements that we believe are needed to promote transfer of learning. Results provide initial evidence that the mixed-training AT can generalize to speech in competition outcome measures beyond the active control condition. The results from this early-stage effectiveness study in individuals with no known hearing difficulties sets the ground for future research to determine the possible effectiveness of Listen for populations with hearing difficulties, as well as mechanistic studies to determine the extent to which the different ingredients of the mixed training contribute to the AT outcomes and further definition, expansion, and refinement of the hypothesized basis sets tested here.

## Methods

### Participants

Fifty-four undergraduate students (47 female, *M* age = 20.8 years, *SD* = 3.24 years) from the University of California, Riverside, were recruited for participation in the study. All participants reported having no difficulties with their hearing or vision, and no history of psychiatric or neurological disorders, and provided signed informed consent as approved by the University of California, Riverside Human Subject Review Board. They received course credit for their participation. Because the data collection took place during the COVID-19 pandemic, testing was administered remotely in participants’ homes via video calls and using their own equipment (e.g., computer or tablet and headphones). Because this was a fairly lengthy study—37 sessions—and data collection took place during the summer months when the COVID-19 infection rate was on the rise, it was challenging to recruit participants and there was significant attrition, with 21 participants leaving the study before its completion. An additional three subjects were excluded due to incomplete data sets caused by administration errors. Thus, the data presented represent the 30 remaining participants who completed all test sessions divided in two groups, the mixed training group (13 female, *M* age = 21.26 years, *SD* = 4.25 years) and the frequency discrimination control group (12 female, *M* age = 21.06 years, *SD* = 2.43 years) further described below.

### Materials

Participants used the hardware that was available to them (most commonly iPhones) as well as the headphones of their choice (most commonly Apple AirPods) and were asked to use the same combination of device and headphones for the entire study. In this aspect, this is an effectiveness study of auditory training which embraces the diversity in technological systems (e.g., tablet and headphones) and environmental conditions (see Green et al., 2019) as features that allow us to determine the extent to which the AT will be effective in ecological conditions (e.g., what could be expected from people downloading and using the training program in their individualized ecological conditions).

### Minimum Audibility

All participants were able to respond to the stimuli, thus ensuring minimal audibility. Because participants were using their own equipment and testing took place remotely, we were not able to calibrate the devices and so the exact presentation levels are unknown. To address this, signal audibility was assessed in two ways that are common in the practice of audiology—2-kHz pure tone detection and single-talker sentence detection. Gallun et al. (2018) showed that the single-talker sentence detection task in PART correlates well with speech detection thresholds tested clinically. For ease of reference, levels are specified in nominal dB, which refers to the level that would have been obtained in an acoustical system consisting of a calibrated digital-to-analog converter and set of electroacoustical transducers (such as headphones) to which the same digital signal was applied. Our experience is that uncalibrated Apple iOS devices are typically within a few dB of their calibrated equivalents.

While there were several participants with surprisingly high detection thresholds on both the tone and speech audibility tasks (see Supplement Figure Sc1), they were still able to perform the training task, suggesting that the high detection thresholds represent motivational lapses, or distractions in within their testing environments (a topic of relevance for further research and approaches to control), rather than poor audibility that might occur due either to hardware incapable of producing the range of sounds needed, or to listeners incapable of detecting the sounds used in the training. For this reason, none of the participants were excluded from the study on the basis of detection thresholds.

#### 2-kHz Pure Tone Detection in Quiet

Participants were asked to indicate if they heard a 100-ms, 2-kHz pure tone presented diotically (to both ears). Presentation level started at a nominal level of 70 dB. Following three consecutive “yes” responses, indicating the detection of the tone presented, the presentation level of the tone decreased first by a step of 20 dB, then in steps of 10 dB until a presentation level of 10 dB was reached, at which point presentation level decreased in steps of 5 dB until a value of 0 dB was reached or three consecutive ‘no’ responses were recorded. The level with the last correct response made was registered as threshold. Participants were able to detect the tone at presentation levels under 30 dB on average (*M* = 21.16, *SD* = 13.9). This suggests both that the hardware used was capable of presenting soft sounds and that the listeners were generally able to detect those soft sounds.

#### Single-Talker Speech Identification in Quiet

Sentences from the Coordinate Response Measure corpus (CRM, Bolia et al., 2000) produced by a single talker were presented (e.g., *Ready Baron go to blue six now.*) and participants were asked to correctly identify which combinations of four possible color and eight possible number keywords they heard. Responses were made on a 4 × 8 a grid of color-number combinations. Presentation level started at a nominal level of 60 dB. The level was decreased by 5 dB after every three trials until 2 out of three responses at a given presentation level were incorrect which ended the task. Participants were able to perform under 40 dB on average (*M* = 36.83, *SD* = 8.5), again suggesting that the hardware and listeners were performing within the expected range.

### Procedure

This study is considered a double-blind randomized actively-controlled study, as both research assistants and participants were blind to the fact one condition was designed as a control for the learning hypothesis behind the other condition (see Green et al., 2019). The study began with an initial enrollment in which participants completed their informed consent forms, were informed of the experimental schedule, demographic information was collected, and device and headphones type they were planning to use were noted. Participants were then randomly assigned either to the mixed (experimental) training condition or to the frequency discrimination (control) training. After attrition, fifteen participants completed the study in the mixed-training condition and another fifteen participants finished the control condition.

**Fig. 1:**
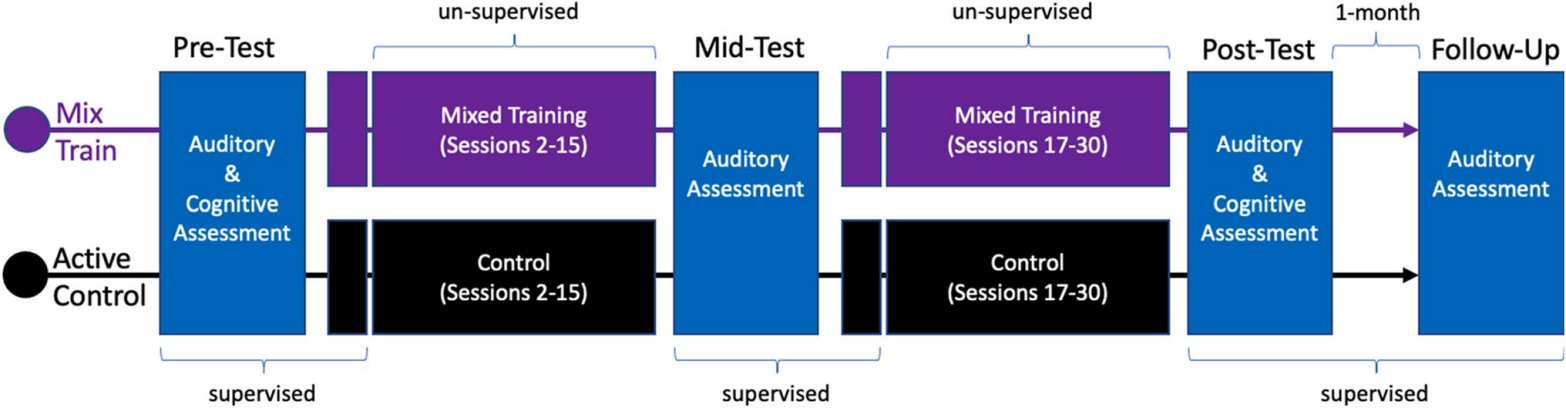
Schematic of the procedures of each training group. Supervised assessment sessions of central auditory or cognitive processing are shown in blue. Training is shown in purple for the mixed-training and black for the active control. The first and 16th session of training were also supervised. Follow-up assessments were conducted 1 month after the last session

Following enrollment, each participant was asked to complete a total of 38 sessions: divided in 30 training sessions and 8 assessment sessions. The assessment conditions consisted of three pre-test, one mid-test, three post-test and one follow-up session that was conducted approximately one month after training (see Fig. 1). The three pre-test sessions were monitored via video using internet-capable video calling software. The first pre-test session consisted of an audiologic case history, the minimum audibility assessments, and the speech in competition assessments (about 30 minutes). The second pre-test session consisted of the rest of our supra-threshold hearing assessments (about 36 min). In the third pre-test session, participants completed the cognitive assessments (about 25 min) as well as the first session of training (25 min). The assessments will be described in detail below.

After the pre-test sessions, participants completed their first session of training with supervision and were asked short questions to assess initial expectations. After this, they were asked to complete two unsupervised training sessions per day (25 minutes each) on seven days for another 14 sessions of training. There was a lockout that ensured participants did not do more than two sessions every 24 h and that participants delayed no more than one week so that this first phase of training would conclude in no longer than two weeks. Then, the mid-training assessments were applied and monitored via video (minimum audibility and speech in competition tests; 25 min). In this same session and after a short break, participants completed their 16th session of training. Following this, participants trained at their homes the recommended two sessions per day (25 min each) for the remaining unsupervised training sessions. After this, participants completed the post-training assessment sessions which were organized identical to the pre-training assessment sessions and were monitored via video. About a month after all the post-tests were completed, a video-monitored follow-up session was carried out; this session was identical to the mid-training assessment session and contained only minimum audibility and speech in competition assessments.

### Training

**Fig. 2:**
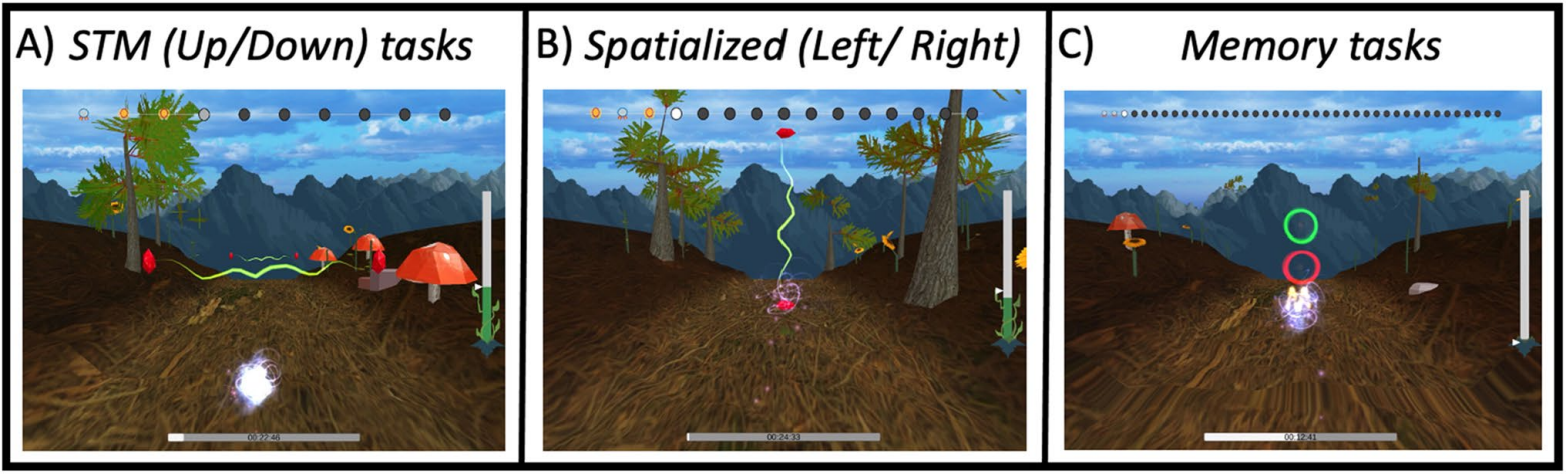
Screenshots of the game Listen in its three main task categories: the STM up/down tasks, the spatialized left/right tasks and the memory tasks

In the game experience, players controlled a game avatar (the “wisp”) that stayed in the center of the screen while the landscape’s optic flow suggested movement towards it, giving the impression that the wisp was flying through the landscape (see Fig. 2). Players were asked to help the wisp avoid obstacles or choose from among options based on a variety of sound cues. Correct responses made the wisp avoid obstacles and absorb energy from the environment, while incorrect responses made the wisp crash into obstacles and lose energy. Both the positive and negative energy effects were accompanied by auditory feedback that indicated whether participants made a correct or incorrect response. The difficulty of the task adapted along a single parameter depending on these responses. As players made progress through the game, new levels with new sounds were unlocked and difficulty related to sound processing was increased along a number of parameters associated to task types as detailed below.

All trials (obstacles) were presented in “streaks” of varying size. Within a streak, the adaptive parameter was not changed. The number of streaks was determined for each task type in the beginning of a “run” depending on the game’s progression logic and was displayed in the upper section of the screen as nodes to be filled up with medal-like or red cross tokens depending on within streak proficiency of performance. The number of trials within each streak was equal to the number of correct responses in the prior streak plus one, with a minimum of one and a maximum of five. After each streak, if every trial within the streak received a correct response, then the adaptive parameter was stepped down by a magnitude specified for each task type below. If fewer than 75% of the trials received a correct response, then the adaptive parameter was stepped up a number of times equal to the number of errors made within the streak, otherwise the adaptive parameter remained unchanged. A status bar on the right side of the screen indicated proficiency of performance within a run (see Fig. 2). Once all streaks in a run were finished, the next task was selected randomly from the pool of available unlocked tasks. A timer was displayed at the bottom of the screen that indicated the time remaining in the given session. Training sessions ended once both the timer reached zero and the current run was completed.

**Fig. 3:**
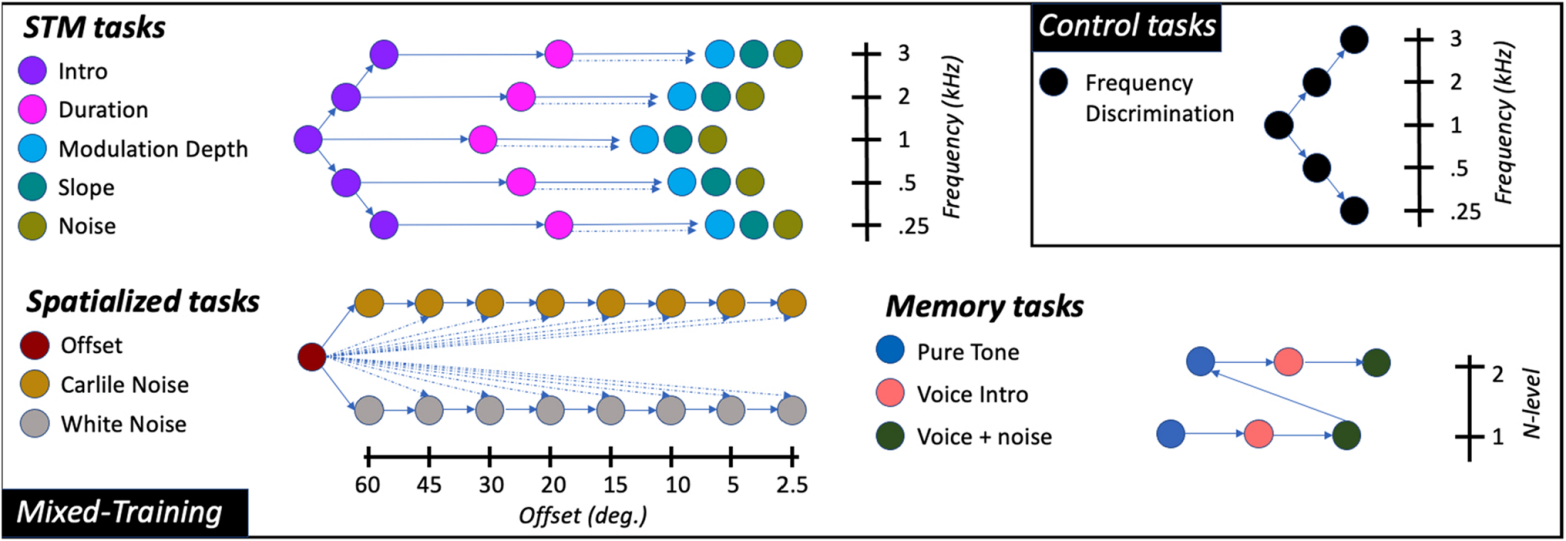
Schematic of the tasks and progression for the mixed-training and active control. Different task types are presented in different colors and are grouped in three categories (e.g., left/right). Solid arrows show progression based on some level of performance. Dotted arrows indicate additional conditional relations (see Supplement). Each of the different task types adapts on a single perceptual parameter (usually name of task). Up/down category tasks are further divided in five target center frequencies (so is the control). Left/right category, noise type tasks are further divided in fixed offset-from-center versions. Memory tasks are further divided depending on memory load. The control condition is shown in the top right panel; this tasks adapts separately on each tone frequency

1. Active Control: Frequency Discrimination Training
  Frequency discrimination training contained the same visual landscape and positive and negative feedback described above and the task required participants to avoid obstacles by swiping upward or downward on the touchscreen to indicate whether a test frequency associated with the obstacle was higher or lower, respectively, than a target frequency that was presented slightly before the test frequency. Target frequencies were centered at 250 Hz, 500 Hz, 1 kHz, 2 kHz or 3 kHz with a random rove of 15% around the center frequency to prevent sensory adaptation. The adaptive parameter of this task was the frequency ratio between target and test frequencies. As participants made progress, the frequency ratio decreased from a value of 0.5 (frequency difference is equal to half the target frequency) towards zero (no difference) with a minimum value of 0.001 and a maximum of 1.
2. Experimental (Mixed) Training
  Mixed-training differed from the active control frequency discrimination task in that it contained three different task categories: up/down STM discrimination; left/right spatial discrimination, and auditory memory (see Figs. 2 and 3). The task conditions, stimuli, and progression logic are described schematically in Fig. 3, with the full details provided in the supplementary materials (Section SA). All these tasks were presented both in quiet and with competing background noise. Competing noise was either broad-band white noise or "Carlile" noise (Carlile & Corkhill, 2015), which is created by vocoding speech into 22 bands spaced on an equivalent rectangular bandwidth (ERB) scale from 50 to 16.5 kHz, and then temporally offsetting each band by rotating randomly in a circular buffer. Carlile noise thus contains the long-term spectrum and within-band amplitude modulations of speech but is unintelligible.

  STM Tasks
    The STM up/down tasks required the participant to swipe upwards or downwards to help the wisp move up or down to avoid a horizontal obstacle, as shown in Fig. 2. The cue provided was a narrow-band spectro-temporal modulated noise with one octave bandwidth centered around one of five different frequencies: 250 Hz, 500 Hz, 1 kHz, 2 kHz, and 3 kHz with a random rove of 15% around the center frequency. The additional acoustical details of these stimuli are as described below in the STM discrimination assessment. This category of tasks started with the *Intro* task type with a center frequency of 1 kHz. In this *Intro* task, an additional frequency-modulated (FM) sweep was presented with the STM narrow-band stimuli to help the listeners learn how to move the wisp up or down in space in response to a stimulus that moved up or down in frequency. The FM sweep adapted on a sweep-to-STM level difference with a step size of 5 dB, from -20 dB where only the FM sweep is presented, to + 20 dB where only the STM stimulus is presented. After completing this *Intro* task, new frequencies for the Intro task type were unlocked as well as the *Duration* task type with 1 kHz center frequency as shown in Fig. 3.
    *Duration* task types adapted on the duration of the STM sound with a step factor of 1.05, an initial and maximum value of 500 ms, and a minimum value of 60 ms. The temporal modulation rate scaled such that one complete temporal cycle was always completed over the duration of the stimulus. When participants reached a duration of 300 ms with their performance, the *Depth*, *Slope*, and *Noise* task types would unlock with a fixed duration of 300 ms. *Duration* tasks remained available in the pool of task types and when a performance value of 60 ms was reached for a given center frequency, a harder version of *Depth, Slope*, and *Noise* tasks with 60 ms fixed stimulus duration was unlocked.
    *Depth* task types adapted modulation depth on an exponential scale with a step factor of 1.2, from 40 to 0.01 dB. The *Slope* task types adapted on the percentage of a complete cycle that was completed over the duration of the stimulus and adapted using a step factor of 1.1, from 1.0 to 0.01 cycles. Finally, the STM *Noise* task types presented white noise in competition with the STM stimulus and adapted on noise-to-signal ratio with a step size of 2 dB, from -20 dB to + 30 dB. At the extrema of the range (-20 dB, + 30 dB), only the louder stimulus was presented.
  Spatialized Tasks
    The Spatialized left/right tasks required the participant to swipe leftwards or rightwards on their touchscreens to help the wisp move left or right in visual space to avoid vertical obstacles in response to a stimulus that was presented to the left or right of midline in auditory space. Stimuli were 240-ms long synthetic vowels—/a/, /ae/, /i/, and /u/—generated with a Klatt speech synthesizer implemented through Praat (Boersma and Weenink, 2014), using a 44.1 kHz sampling rate and were low-pass filtered at 5 kHz. Onset and offset ramps were 20 ms. Different task types adapted on either spatial offset or noise level. This category of tasks started with the *Offset* type where the stimulus is adapted on angular offset from center with a step factor of 1.1, starting from 60 degrees and down to 0.1 degree. Depending on the value reached in this *Offset* task, the *White Noise* and *Carlile Noise* tasks would be unlocked at a fixed offset of 60 degrees. Spatialized *Noise* task types presented noise spatialized forward adapting on the noise-to-signal level with a step size of 2 dB between -20 dB and + 20 dB. At the extrema (-20 dB and + 20 dB), only the louder stimulus was presented. Achieving a noise-to-signal performance level of 0 dB unlocked the next fixed offset (e.g., 45 degrees) for the *Noise* tasks if that offset had already been unlocked from the *Offset* task.
  Memory Tasks
    The Memory tasks required the player to swipe upwards or downwards to help the wisp choose between the two rings presented instead of obstacles (shown in the far right panel of Fig. 2). This task did not use the streaking mechanism and each trial was evaluated individually in the staircase. When the rings appeared, the player was required to compare the sound just heard with one stored in memory. In the “1-back” condition, the wisp needed to fly through the top green circle if the last sound matched the one before it, or through the bottom red circle if the sound did not match the one before. In the “2-back” condition, the comparison was to the sound that had been played two before it, representing a greater memory load. If there was not a match, the player was to direct the wisp through the bottom red ring. The sounds to be memorized were distributed in three task types: *Pure Tone* using sinusoidal tones, *Voice Intro* using synthetic vowels in quiet, and *Voice* + *Noise* which used vowels in competition with white noise. Progression occurred from simpler sounds towards more complex and the “2-back” conditions were only unlocked for each task type after a 90% accuracy of performance was reached for the “1-back” conditions. Once the *Voice* + *Noise* task “2-back” condition was achieved, this would be the only memory task available for training.

### Assessments

All participants completed the same assessments before, in the middle, and after training. Assessments were carried out remotely using applications developed at the BGC: PART for the auditory perception tasks and Recollect (https://braingamecenter.ucr.edu/games/recollect/) for the cognitive processing measures, also available online. The assessments were organized into three groups: speech in competition assessments (primary outcome), basic auditory tests of supra-threshold hearing, and cognitive assessments. The speech in competition assessments included tests of spatial release from masking and identification of spoken digits in noise, and were carried out at pre-, mid-, post-training, and follow-up time points. The basic supra-threshold auditory tests included dichotic FM, gaps-in-noise, and spectro-temporal modulation detection and discrimination tests. These basic supra-threshold tests were assessed only at the pre- and post-training time points. The cognitive assessments included spatial working memory, working memory updating, countermanding, and cancellation tests, and were also only applied at pre- and post-training time-points. This design reflects our interest on the speech in competition measures as primary outcome measures with the other measures considered to be secondary/exploratory outcomes.

1. Assessments of Speech in Competition
  a Spatial Release from Masking
    Identification of speech targets in the presence of two competing speech maskers was measured using a method developed by Marrone et al. (2008) and modified by Gallun et al. (2013). Two conditions were tested: one in which all three talkers are presented with the same interaural differences (*colocated*) and one in which the target appears to be located in front of the listener and the maskers are located to the left and right of center with an offset of 45 degrees (*separated*). All spatial locations were simulated over headphones by convolving the speech stimuli with the appropriate head-related impulse responses for each location as described in Gallun et al. (2013). Target level was fixed at a nominal level of 65 dB and the level of each masker was progressively increased after every two responses, starting at a target-to-masker ratio (TMR) of 10 dB and progressing over 20 trials to a TMR of -8 dB. Threshold is estimated based on the total number of correct responses as described in Gallun et al. (2013). Speech stimuli were taken from the same CRM corpus that was used for the speech audibility pre-test, described above. On each trial, as in the pre-test, participants identified color/number combinations uttered along with the call-sign “Charlie” by one of three male speakers. In this case, however, the target was presented in competition with two other male speakers uttering different color/number/call-sign combinations from the CRM sentences. The color/number combination was identified by clicking on a color/number grid presented on screen. The dB difference between TMR thresholds in the colocated and separated conditions is used as a Spatial Release from Masking metric and reflects the ability to benefit from spatial cues.
  b Digits in Noise Identification
    The targets in this task were digit triplets spoken in competition with white noise (Smits et al., 2013) and presented in a 25-trial 1-up/1-down adaptive staircase where the presentation level of the target decreased by 2 dB following a correct response and increased by 2 dB following an incorrect response. Both target and noise started at a nominal level of 70 dB, and the noise level was held constant for all trials.
2. Basic Supra-threshold Auditory Assessments
  These tasks employed a 4-interval, 2-cue 2-alternative forced-choice format as described in Larrea-Mancera et al. (2020) where four squares were presented on screen and lit up sequentially in coordination with four auditory intervals. The first and last intervals always presented *standard* stimuli (thus referred to as *cue* intervals), in contrast to the two alternatives in the middle intervals, one of which would match the cues and the other would contain the *target* of interest. The target would differ from the standards based on a single parameter, which would be adaptively varied based on performance. Adaptive tracking involved two-stage adaptive staircases with a 2-down/1-up rule, meaning that two correct responses would make the task harder and one incorrect response would make it become easier. The first stage of the staircases contained three reversals and had step sizes five times larger than in the second stage, which contained six reversals. Thresholds were calculated from the average of the second stage reversals. Step sizes of the staircases were kept at a ratio of 1:1.5, which indicates that the step up was 1.5 the size of the step down. Further details of the staircase parameters are given for each task below.
  a Dichotic FM Detection
    The stimuli were those used by Larrea-Mancera et al. (2020), based on the dichotic FM detection task developed by Green et al. (1976) and modified by Grose and Mamo (2010, 2012) and Hoover et al., (2019). Standard intervals contained pure tones with a frequency drawn at random from the range 460–540 Hz. Each was 400 ms in duration and was presented at a nominal level of 75 dB. Onset and offset ramps were 20 ms. Target intervals contained tones drawn from the same frequency range, the same level and the same duration as the standard intervals but had an anti-phasic 2-Hz frequency modulation across left and right ears (dichotic). The target interval adapted on modulation range (Hz) on an exponential scale starting at 10 Hz and stepping down by 2^1/2^ Hz for the first stage and 2^1/10^ Hz for the second with a minimum value of 0 and a maximum of 10 kHz.
  b Gaps-in-Noise Detection
    This assessment involved the use of a noise stimulus upon which are imposed brief silent gaps, the detection of which requires temporal processing of envelope and temporal fine structure cues (see Grose et al., 1989; Florentine et al., 1999; Hoover et al., 2015, 2019). Standard intervals were 400-ms long white noise presented at a nominal level of 70 dB. Onset and offset ramps were 20 ms. Targets were the same noise stimuli into which a brief silent gap had been introduced. Across trials, gap duration (ms) was adaptively varied on an exponential scale starting at 20 ms and stepping down towards zero by 2^1/2^ ms for the first stage and 2^1/10^ ms for the second with a maximum value of 60 ms.
  c Spectro-Temporal Modulation Detection
    The STM stimuli used were from Larrea-Mancera et al. (2020). Standard intervals were 300 ms white noise from 400 Hz to 8 kHz presented at a nominal level of 70 dB. Onset and offset ramps were 20 ms. For the detection task (labeled simply STM), targets contained a spectral modulation of 2 cycles per octave and a temporal modulation rate of 4 Hz. Thresholds were measured in terms of modulation depth (dB) which was adaptively varied using a logarithmic amplitude scale measured from the middle to the peak of the amplitude range as described in Stavropoulos et al. (2021) as M (expressed in dB). Adaptation started at 6 dB and stepped down by 0.5 dB for the first stage and 0.1 dB for the second with a minimum value of 0 and a maximum of 10 dB.
  d Spectro-Temporal Modulation Discrimination
    For the STM discrimination tasks (labeled STM_250 and STM_3k), STM stimuli were presented in all four intervals, but the direction of modulation for one of the stimuli in the second and third intervals matched the modulation direction (up or down) in the first and fourth intervals (the standard “cues”), while the other did not. To make the task more difficult, a narrowband noise (1 octave wide) was also presented on each interval. In one task, the targets and maskers were centered at 250 Hz and in a second task, all were centered at 3 kHz. Performance was measured by adaptively varying the modulation depth, starting at 10 dB and stepping down by 2^1/2^, every three trials until 4 or more errors were made in the last 6 trials.
3. Assessments of Cognitive Processing
  The cognitive assessments were selected to represent measures of general domain cognitive processes thought to be related to perception and include measures of working memory, attention, and inhibition.
  - Spatial Working Memory (Corsi blocks)
    This task, originally developed by Corsi (1972), relies on accurate sequential storage and retrieval of sequences in working memory. An array of squares (drawn to represent gopher holes) is distributed asymmetrically in space and presented to subjects. In this modified version, for every trial, gophers come out one at a time from holes already present on the screen (traditionally squares are pointed to or change color in computer versions). Gophers are visible for 1.5 s (0.25 s rising from the hole, 1 s waiting above the hole, 0.25 s descending into the hole) in a random sequence with inter-stimulus-intervals (ISIs) of 0.5 s. Participants had to identify the holes where the gophers were presented in the order in which they had appeared. Participants had 10 s to respond. After every response, the next trial started after an inter-trial-interval (ITI) of 1 s.
    Every time a sequence of holes was identified correctly, the number of elements in the sequence increased, starting with two-element sequences and progressing towards a maximum of ten-element sequences. When an incorrect response occurred, the number of elements in the sequence would not change. If a second incorrect response occurred, the number of elements decreased by two but never went below two. The second time two incorrect responses were provided in a row, the test would end. Span scores were computed the longest sequence achieved with at least one correct response.
  - Working Memory Updating (n-back)
    Similar to the memory task used in the mixed-training, we used an n-back task (Kirchner, 1958; see Pergher et al., 2020) in which participants were required to report what they saw (rather than what they heard) n-items back in a continuous, sequential presentation divided in 5 blocks. On each trial participants had 2500 ms to respond if the presented animal cartoon (e.g., sheep) matched (or not) the animal presented “n” (load) trials back with an inter-stimulus-interval (ISI) of 500 ms. We first presented 29 + n trials of the 1-back, then we presented a block of 9 + n practice trials of the 2-back followed by a block of 29 + n trials of the 2-back, then a block of 9 + n practice trials of the 3-back followed by a block of 29 + n trials of the 3-back. Accuracy was calculated for each of the n-levels from the number of hits divided by the sum of hits, misses and false alarms. Correct rejections did not contribute to accuracy scores.
  - Countermanding
    This task provides a measure of cognition additional to those of working memory related to inhibition. It is based on Wright and Diamond (2014) but uses dogs and monkeys instead of hearts and flowers for congruent and incongruent stimuli. On each trial, two buttons were presented on the sides of the screen. Atop one of them, one of two stimuli was presented. A picture of a dog required the participant to press the button on the side of the screen with the picture. A picture of a monkey required the participant to press the button on the other side. Participants were instructed to respond as fast as they could. The key process is that participants need to inhibit one stimulus–response relation to act on the other. After a short introduction of three trials, a dogs-only condition was tested for 12 trials. Then monkeys were introduced for three trials and tested for 12 trials. After this, a mixed condition with dogs and monkeys is introduced for three trials and then tested for 48 trials. Reaction times constitute the main outcome measure of this test.
  - Cancellation
    This is a test of selective and sustained attention that resembles the D2 test (Brickenkamp & Zillmer, 1998) where participants are instructed to sequentially search and mark a set of target items in a series of similar items. In our variant called UCancellation, participants were presented sequentially with visual targets in the form of dogs and monkeys that varied in their orientation (facing right or left) and color distribution (same color palette). Participants had to select a target type of dog/monkey among distractors with similar features and colors. Eight pictures were displayed per row, with 3–5 targets per row; every 10 rows had exactly 40 targets. Each row was displayed for a maximum time of 6 s (with 1 s blank screen interval between rows). One auditory cue signaled that time had run out for a particular row and a different auditory cue was presented if the participant cancelled all targets in a row with no false alarms. Participants completed a short practice run of about 10 trials and then were tested for 3 min and 30 s. Scores were computed out of the number of hits minus the number of false alarms.

### Data Analysis

Data analyses were organized around two main questions: 1) Was there an improvement in the outcome measures collected within the groups from the Pre-Test to the Post-Test? 2) Are any improvements found greater in the experimental group compared to the active control? For the first question we conducted related-samples t-tests between pre- and post-test scores within each group. For the second question we conducted independent-samples t-tests on the difference between pre- and post-test scores (Pre–Post) of each group, which is equivalent to the interaction term of a mixed model 2-by-2 ANOVA. Based upon the a priori hypothesis that the mixed training would lead to greater positive changes on speech in competition tasks than the active control, one-tailed tests were conducted for the speech outcome measures. Given that there are multiple measures of the same constructs (as recommended by Simons et al., 2016), and to minimize multiple comparison issues, we computed composite scores based on the following groupings: *Basic Auditory Composite:* gap in noise, dichotic FM, and the STM; *Speech in Competition Composite:* spatial release from masking tasks (colocated and separated conditions) and the digits in noise task; and the *Cognitive Composite:* spatial working memory, working memory updating, countermanding and cancellation.

## Results

The results are presented in three sections: training data, auditory perceptual outcomes, and cognitive outcomes.

### Training Data

Because the training was designed to give participants experience across a range of hearing dimensions, it is difficult to compute a simple measure that captures overall performance. However, one way to understand training performance is to examine the extent to which participants progressed across the task matrix presented in Fig. 3. Further results are described in the Supplemental Materials section SB. All individual runs for all tasks used in both training conditions are shown in figures Sb1 to Sb10.

All participants in the mixed-training group made substantial progress through the game’s different levels. In the case of Spatialized (left/right) tasks, all participants made progress in terms of the offset from center where targets were presented from the highest magnitude of 60 to below 2.5 degrees (see Fig. Sb2 in the supplement). Likewise, in the case of the Memory tasks, all participants progressed to the final 2-back task achieving average SNR thresholds of approximately -4 dB. In the case of the STM discrimination (up/down) tasks, all participants progressed out of the intro layer of tasks and unlocked the duration adaptive layer across all five center frequencies tested. Only two-thirds of the participants unlocked STM discrimination tasks that adapted on either depth, slope or in terms of competition with noise.

In the control training, which involved fewer conditions, participants quickly unlocked all tasks with the five center frequencies tested (see Fig. Sb1 in the supplement) and achieved average thresholds that were less than 0.05 of the center frequencies tested.

### Auditory Perceptual Outcomes

Table 1 shows mean pre- and post-training performance on assessments for both groups. At baseline, mean performances of the experimental and control groups were similar (within half a standard deviation) to thresholds previously reported for remote testing in a similar sample (Larrea-Mancera et al., 2021) in the dichotic FM assessment (*M* = 0.82, *SD* = 2.48), the STM assessment (*M* = 1.24, *SD* = 0.61), and the speech-on-speech masking tasks in the colocated (*M* = 2.89, *SD* = 1.58) and separated conditions (*M* = -1.81, *SD* = 3.68) as well as in the spatial release from masking (SRM) metric (*M* = 4.43, *SD* = 3.38). These data suggest that participants overall performance on auditory tasks was within what would be expected based on previously obtained norms. Table 1 also presents the comparisons of training-related change within each group for exploratory purposes only as the main analysis is based on composite scores. Table 2 presents the comparisons between the change (difference) scores obtained in each group also for exploratory purposes only.

**Table 1:**
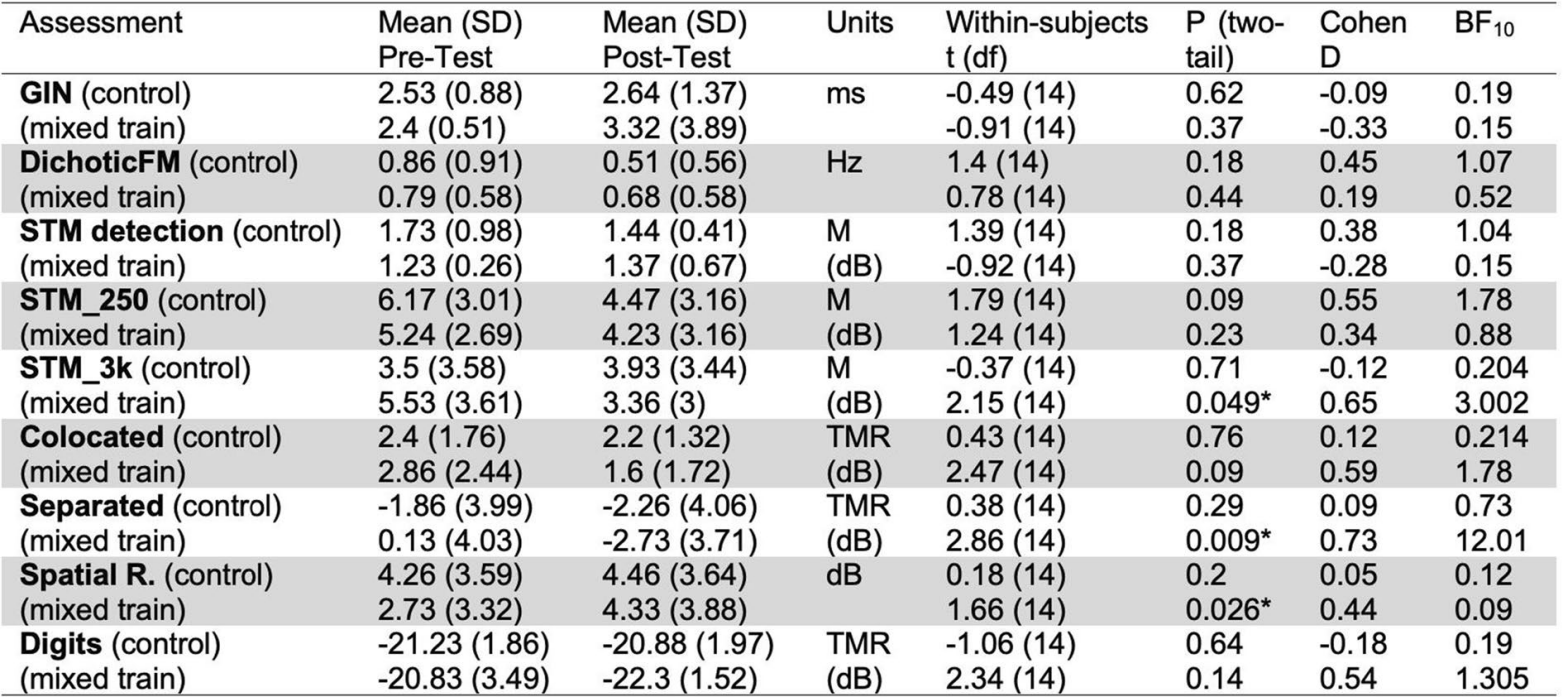
Statistics for each of the auditory assessments addressing within-group training-related change. Related-samples *t*-tests (frequentist and Bayesian) are also provided

**Table 2:**
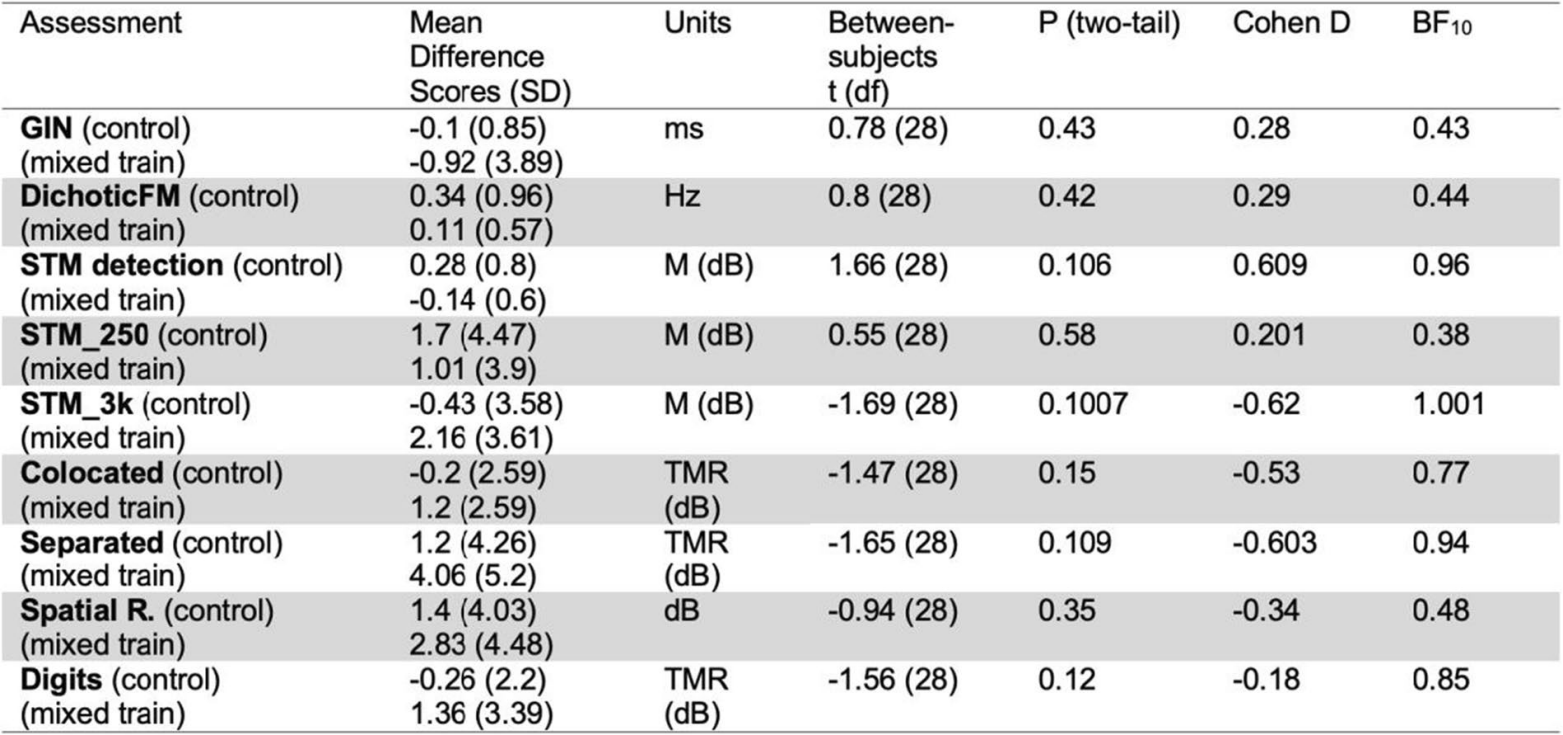
Statistics for each of the auditory assessments addressing between-group training-related change using difference scores (pre–post). Independent-samples *t*-tests (frequentist and Bayesian) are also provided

**Fig. 4:**
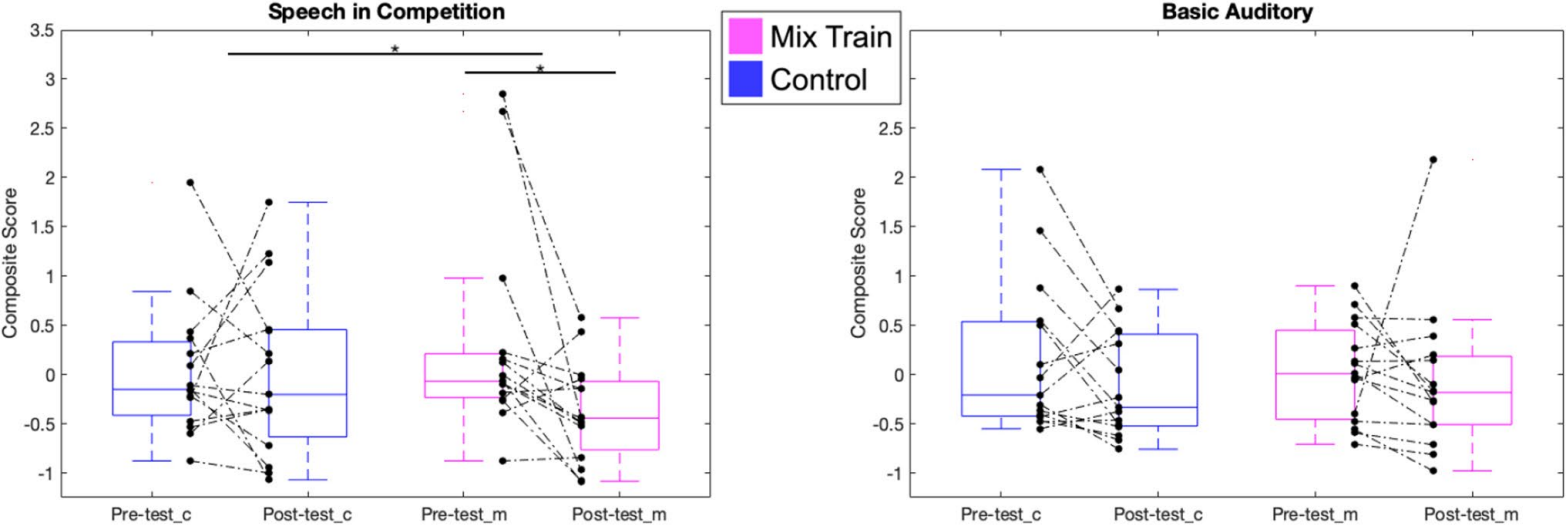
Data from pre- and post- Composite Measures of hearing. Blue boxes show Control group (_c) data and magenta boxes the mixed-training group (_m). Black dots indicate individual thresholds and dotted lines the individual trajectory of performance change (pre to post)

To address the primary training outcome, namely the effectiveness of the AT approach to promote transfer to improved performance on measures of speech in competition, we examined changes across time on speech in competition composite score (see Fig. 4) that consisted of the colocated and separated measures from the spatial release from masking tasks and the digits in noise measure (individual task statistics are shown in Table 1). This composite had a strong internal reliability at pre-test across both groups (*Cronbach’s alpha* = 0.79) which indicated this composite is suitable to represent the assessments it contains. For this measure we observed a significant improvement for the mixed-training group (*t*_*(14)*_ = 2.61, *p* = 0.01, *Cohen’s d* = 1.19) but not for the control group (*t*_*(14)*_ = 0.05, *p* = 0.47, *Cohen’s d* = 0.01). Importantly, there was also a significant difference in the change scores between groups (*t*_*(28)*_ = -1.91, *p* = 0.033, *Cohen’s d* = -0.68), showing that the improvement in speech in competition composite was significantly greater than that of the control group. These results provide preliminary evidence that the mixed-training may provide benefits to tasks of speech in competition, however we note the small sample size and that the effect would not pass a two-tailed test, and so it will be important to replicate these results.

To explore whether the AT led to changes in other supra-threshold hearing assessments, we examined a basic auditory processing composite (see Fig. 4). This composite also had strong internal reliability at pre-test across both groups (*Cronbach’s alpha* = 0.73) which indicated this composite is also suitable to represent the assessments it contains. For this measure, we observed no statistically significant changes in either the mixed-training group (*t*_*(14)*_ = 0.44, *p* = 0.66, *Cohen’s d* = 0.11), nor the control group (*t*_*(14)*_ = 1.57, *p* = 0.15, *Cohen’s d* = 0.39). Further, an independent samples t-tests on these difference scores (mixed-training vs control) revealed no statistically significant differences in the basic auditory composite (*t*_*(28)*_ = 0.63, *p* = 0.54, *Cohen’s d* = 0.22), between the training groups.

4. Dosage and Retention Effects

**Fig. 5:**
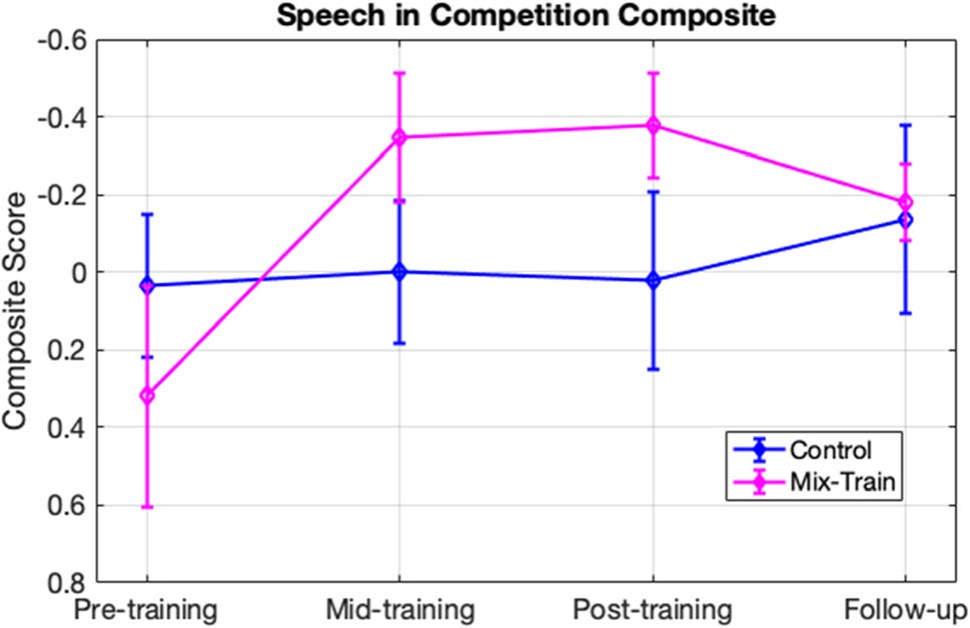
Shows the average thresholds for the speech in competition composite before, during and after training including a 1-month follow-up. Error bars represent standard error of the mean

To address how much training was required to achieve the observed improvement on the speech in noise tests, we examined data in the mid-test. First, addressing the issue of dosage, we observed an improvement on the speech in competition composite when comparing the pre-test to the mid-test (*t*_*(28)*_ = -2.47, *p* = 0.01, *Cohen’s d* = -0.88). Next, we examined whether learning was retained after an interval of one month without training. We did not find statistical evidence in support of a benefit from pre-test to follow-up (*t*_*(28)*_ = -0.96, *p* = 0.17, *Cohen’s d* = -0.34). The difference found in thresholds between pre-test and mid-test in the mixed-training group appears to be no different than that of pre-test to post-test (*t*_*(14)*_ = -0.25, *p* = 0.8, *Cohen’s d* = -0.06), suggesting that 15 sessions is a sufficient dose of training, however data from the follow-up fails to show that effects remain the same across time, at least for normally hearing young adults used in the present study (Fig. 5).

### Cognitive Outcomes

**Fig. 6:**
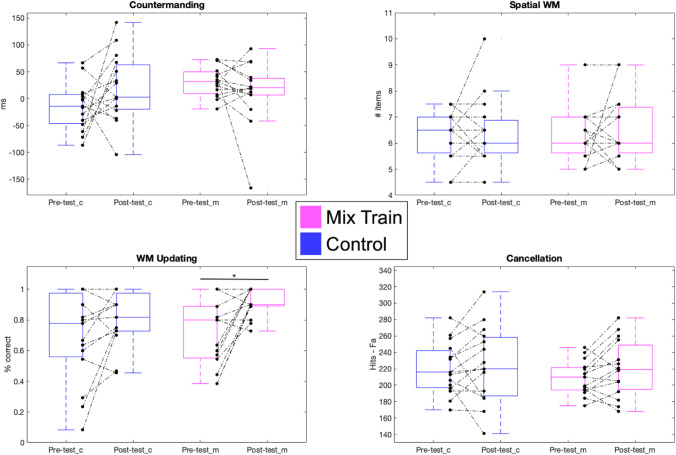
Data from pre- and post- measures of cognitive processing. Blue boxes show Control group (_c) data and magenta boxes the mixed-training group (_m). Black dots indicate individual thresholds and dotted lines the individual trajectory of performance change (pre to post)

The cognitive composite had only a moderate internal reliability at pre-test across both groups (*Cronbach’s alpha* = 0.41) which indicated this composite is probably not the best way to represent the assessments it contains. After this analysis, it was clear that different tasks either explain different aspects of the variance, or perhaps some of them were unreliable, thus spreading noise through the rest of the measures, and so the cognitive composite was not used to evaluate training outcomes. Instead, each assessment was examined separately (see Fig. 6). For the countermanding test, a conflict score was computed by subtracting the average reaction time for responding to the dogs from the average reaction time for responding to the monkeys. This metric providence no evidence of change in the mixed-training group (*t*_*(14)*_ = 1.21, *p* = 0.48, *Cohen’s d* = -0.306), or in the control group (*t*_*(14)*_ = -1.63, *p* = 0.24, *Cohen’s d* = -0.41). For the spatial working memory span, we did not find significant change in either the control (*t*_*(14)*_ = -0.79, *p* = 0.42, *Cohen’s d* = -0.201) or the mixed-training (*t*_*(14)*_ = -0.89, *p* = 0.76, *Cohen’s d* = -0.22). For working memory updating, performance accuracy on the 1-back was at ceiling and the 3-back at chance performance for most participants, and so we chose to focus on the 2-back. We found accuracy improved significantly for the mixed-training group (*t*_*(14)*_ = -3.74, *p* < 0.01, *Cohen’s d* = -0.94) but not for the control group (*t*_*(14)*_ = -1.96, *p* = 0.069, *Cohen’s d* = -0.49). However, this change from pre to post-test did not differ significantly between the mixed-training and control groups (*t*_*(28)*_ = 1.15, *p* = 0.25, *Cohen’s d* = 0.42). Finally, for the cancellation task we found that neither the mixed-training group showed significant within-group change in scores (*t*_*(14)*_ = -1.82, *p* = 0.089, *Cohen’s d* = -0.45), nor the control group (*t*_*(14)*_ = -0.55, *p* = 0.58, *Cohen’s d* = -0.13). Thus overall, there is little evidence of a reliable change in cognitive measures from this training above the control condition.

## Discussion

This study investigated the effectiveness of a novel gamified approach to Auditory Training (AT) based on neural and cognitive research on speech in competition. Significant improvements were found in the speech in competition tasks relative to those found for an active frequency-discrimination control training. However, no consistent changes were observed in measures of more basic supra-threshold auditory processes. While at first look this may be surprising, it is worth noting that these tasks primary involve detection, rather than the discrimination tasks used in training, and with detection thresholds being superior to discrimination thresholds, the stimulus values in the tests were largely outside of the range presented during the training task, with the exception of the STM discrimination (250 Hz and 3 kHz) tasks, where training improvements were significant or close to significant. Moreover, we did not find significant differences between mixed-training and control groups in terms of the learning effects of AT on the cognitive measures. These results were found in participants who downloaded the software on their own devices and conducted experimental sessions in their own space, suggesting that the results obtained here are similar to what one would expect from a young normal-hearing individual of similar demographics accessing the training tool on their own outside of controlled laboratory settings. We acknowledge the preliminary nature of these results with a small sample size, and plan replication and extensions to other age groups including those different types of hearing loss once human subject research restrictions related to the COVID-19 pandemic are relaxed.

A key question in the literature has been the extent to which expectations may explain effects of cognitive and perceptual training. To address this, we asked participants to report, after their first experience with the auditory training, their expectations regarding whether the auditory training would lead to improvements either on the trained conditions or in other tasks of their daily life using a Likert type scale (1 = Not at all; 2 = Not really; 3 = Can’t say; 4 = Quite a bit; 5 = Very much). Participants neither exhibited strong expectations of improvements on the trained skills (mixed-training *M* = 3.8 *SD* = 0.86; active control *M* = 3.5 *SD* = 0.91), or to untrained activities of daily living (mixed-training *M* = 3.5 *SD* = 0.74; active control *M* = 3 *SD* = 0.75), and there were no statistical differences between the groups (*p* = 0.41 for near transfer and *p* = 0.061 for far transfer) although there was a trend for higher expectation for the transfer to tasks of daily life in the mixed training condition. However, there were no significant correlations between expectations and training outcomes on the speech in noise composite (trained skills, *r* = 0.1, *p* = 0.6; daily life, *r* = -0.064, *p* = 0.73). Thus, we failed to find solid evidence that expectations explained training outcomes of the study, or differences in outcomes between groups.

The effect sizes for some of the speech assessments conducted here are comparable to that of Whitton et al. (2017), which has been heralded as a viable type of AT intervention (see Skoe, 2017), with reported benefits of about 1.5 dB signal-to-noise ratio in a group of people with hearing difficulties. After 15 sessions of training our participants, all of whom reported no hearing difficulties, achieved improvements of a similar size, which did not change with the rest of the training. In our training we found mean differences between pre- and post-training assessments in the speech in competition measures that differ between the mixed training group and the active control by 1.4 dB for the colocated SRM, 2.86 dB for the separated condition and 1.62 dB for the digits in noise test (see Table 2).

It is important to note that the effects observed here were not of a size that reached statistical significance when tested one month after training. This lack of retention leads to the question of whether additional training, or maintenance sessions (e.g., top-up sessions that are shorter and less frequent than full training), could have allowed them to retain these observed benefits. Clarifying the extent to which maintenance training will lead to retention will be a target of our future research. Of note, there is also a question of whether retention may depend on age as in previous studies (e.g., Merzenich et al, 1996; Moore et al., 2005; Tallal et al., 1996) children seemed to retain training for longer periods. Another important future direction will be to test effectiveness of the approach in people of different age groups and with hearing difficulties.

The benefits observed are consistent with Stewart et al. (2020), who suggested that using an action-based video-game that targets auditory cues for its task resolution should yield benefits in the auditory domain. Those authors observed no significant effects after training with an action video-game and suggested this may be due to sensory domain specificity (mainly relying on visuo-spatial cues). Interestingly, we found that the mixed training condition showed significant improvement after training in the working memory updating task (n-back). This benefit was not statistically significant when compared to the active control condition which also showed a tendency for improvement. These results might reflect expected effects from active gamified tasks on WM processes (Deveau et al., 2015) that are thought to mediate auditory processing (Zhang et al., 2016, 2017). It is an interesting question of whether WM updating, or attention switching (Dhamani et al., 2013), are particularly susceptible to training and that they then could underpin improved speech in competition (Gallun & Jakien, 2019). While our findings support the idea that perceptual learning as a result of a gamified AT may transfer to speech in competition measures, we note that, given the complexity of the mixed-training approach (e.g., training multiple stimuli, tasks, and with a complex motivational framework), more research will be required to understand which game elements are of importance to this effect, and how training elements may interact, to promote beneficial change throughout the many brain processes that may be involved in this learning (Maniglia & Seitz, 2018). Possible elements of importance include the motivated engagement characteristic of play behavior (Vygotsky, 1967), the direction of exogenous and endogenous attention (Donovan & Carrasco, 2018; Donovan et al., 2015), the promotion of cognitively challenging “fast activity” (Bediou et al., 2018; Green & Bavelier, 2015), the use of varied stimulus sets (Deveau et al., 2014a, b; Xiao et al., 2008; Zhang et al., 2011), adaptive difficulty ensuring a match of skill and challenge (Ahissar & Hochstein, 1997; Hung & Seitz, 2014), multisensory facilitation of learning (Shams & Seitz, 2008), and the sensorimotor nature of tasks that include a diverse exploration of sensory and motor contingencies (O’Reagan & Noë, 2001; Whitton et al., 2014, 2017). While the distinct elements mentioned here may have specific contributions to perceptual learning and transfer, and there is a need to better understand these contributions to gain mechanistic understanding and improve training design, it is likely they all converge in promoting the learning effects observed to some extent (Seitz & Dinse, 2007), although we cannot rule out some interference (Katz et al., 2014).

There are a number of indications in the literature that our training approach can be improved to boost learning. For example, Whitton et al., (2014, 2017) identified the sensori-motor co-generating element in their “foraging” task, which involved searching for targets with manual movements, as being crucial to promote the effects they have found. Likewise, other studies examining music to promote learning have emphasized this synchronous co-generation of motor behavior and perceptual information (see Zatorre et al., 2007). As our training is an interactive video-game thus already including a series of sensorimotor relations, there is still an opportunity to couple our trained sounds into a co-generative relationship with some of the motor responses they evoke.

Moreover, some have suggested that having a rich multi-sensory training approach might be beneficial to promote learning (Shams & Seitz, 2008) even when the target is unisensory (Shams et al., 2011), as it may benefit from interactions with other sense modalities with different proficiencies (Barakat et al., 2015). Although our training is audio-visual and thus already addresses some of the possible multi-sensory benefit, extensions can be made to integrate additional multisensory cues with visual stimuli that are congruent with the auditory stimuli and can facilitate the auditory stimuli (Seitz et al., 2006; Shams & Seitz, 2008). Future research should explore additional correspondences between visual and auditory cues (see Yehia et al., 2002) and even other senses (see Rosenblum et al., 2017).

A third aspect which could be explored to boost learning is the use of implicit rather than, or in addition to, explicit training. Prior research suggests that implicitly training phonemic categories using temporal synchrony with task-relevant aspects in video-game play may lead to benefits to speech processing (Kimball et al., 2013; Vlahou et al., 2012; Wade & Holt, 2005). Exploring training both under the explicit focus of attention and implicit temporal coupling to task relevant elements outside the focus of attention (e.g., Seitz & Watanabe, 2003; Seitz et al., 2009) may afford more diverse training benefits as different learning mechanisms might be recruited (Seitz & Dinse, 2007; Seitz & Watanabe, 2009).

Notably, given that supra-threshold hearing difficulties differ across individuals, it is likely that more attributes of the training intervention could be personalized to the individual. Our training is designed in such a way that tasks that are difficult for a given individual will remain in the training rotation until the processing precision required by the game to progress to different tasks or difficulties is achieved. In that sense the training is, to some extent, tailored to individual needs, but could still be individualized further. For example, while the frequency-discrimination task is a reasonable control condition for young normally hearing adults, in the case of cochlear implant patients frequency discrimination training directly targets their hearing needs (e.g., Goldsworthy & Shannon, 2014). Thus, for this population there would be important dimensions of hearing to consider (e.g., pure tone discrimination) that might be different than for a population with age-related changes in hearing or for those suffering the effects of traumatic brain injury. Future research with hearing diverse groups of people and across the lifespan is required to further understand what elements of our AT approach may be more important to promote supra-threshold hearing benefits including improvements understanding speech in competition and how this may differ as a function of different individuals’ hearing and listening needs.

Beyond exploring the effectiveness of our AT approach, which represents the main motivation of this study, another matter of interest is of a methodological nature: the extent to which the performance for the different aspects of supra-threshold hearing present in the gamified training match the validated assessments obtained with PART. However, the thresholds obtained during training with similar stimuli to that used for the STM discrimination assessments were of higher magnitude on average (8.23 dB for the 250 Hz and 10.19 dB for the 3 kHz) than the assessment thresholds (see Table 1). Furthermore, there was no relation between the assessment thresholds and the training thresholds for either the 3 kHz center frequency (*r* = -0.001, *p* = 0.9) or the 250 Hz center frequency (*r* = -0.63, *p* = 0.09). Of note, only 9 out of 15 participants in the mixed-training group reached the training task that was equivalent to assessment, making the apparent distance in thresholds even greater. Future work will be required to account for differences in performance between the gamified and non-gamified settings and also which setting may better predict hearing in ecological settings. While the non-gamified testing environment provides a nicely controlled testing environment, the game represents some of the variability of tasks and sounds that are found in ecological settings.

In summary, this study presents a proof of concept that an integral approach to AT that focuses on a basis set of spectral-temporal modulations, sound localization with and without competition, and working memory components can transfer to untrained tasks of speech in competition. Our study demonstrates the feasibility of this dynamical and entertaining game environment to train hearing to be used in participants’ homes and on uncalibrated devices, greatly improving the accessibility and thus potential impact of the approach. Moreover, this study and intervention presents a starting point from which to improve development of auditory training in search for a more optimal learning paradigm. However, a small sample was collected and participants in this study had no reported hearing difficulties. Thus, future research both for replication and extensions to address the extent to which this intervention may provide benefits to people with diverse hearing abilities, and across different age groups that better represent those seeking improvements in their hearing abilities.

## Supplementary Information

Below is the link to the electronic supplementary material.

Supplementary file1 (PDF 5927 KB)

## Data Availability

The dataset generated and analyzed in the current study is available from the corresponding author upon request. The training and assessment programs used are freely available online (see www.braingamecenter.ucr.edu) as detailed in the body of the manuscript.
